# Does the ketogenic diet improve neurological disorders by influencing gut microbiota? A systematic review

**DOI:** 10.1186/s12937-023-00893-2

**Published:** 2023-11-20

**Authors:** Mahdi Mazandarani, Narges Lashkarbolouk, Hanieh-Sadat Ejtahed, Mostafa Qorbani

**Affiliations:** 1 Endocrinology and Metabolism Research Center, Endocrinology and Metabolism Clinical Sciences Institute, Tehran University of Medical Sciences, Tehran, Iran; 2 Golestan University of Medical Sciences, Gorgan, Iran; 3 Obesity and Eating Habits Research Center, Endocrinology and Metabolism Clinical Sciences Institute, Tehran University of Medical Sciences, Tehran, Iran; 4 Non-Communicable Diseases Research Center, Alborz University of Medical Sciences, Karaj, Iran; 5 Chronic Diseases Research Center, Endocrinology and Metabolism Population Sciences Institute, Tehran University of Medical Sciences, Tehran, Iran

**Keywords:** Ketogenic diet, Ketones, Neurodegenerative diseases, Gut microbiota

## Abstract

**Background:**

The aim of this systematic review is to evaluate the changes in gut microbiota (GM) induced by the Ketogenic Diets (KD) as a potential underlying mechanism in the improvement of neurological diseases.

**Methods:**

A comprehensive search was conducted on three electronic databases, including PubMed/Medline, Web of Science, and Scopus until December 2022. The inclusion criteria were studies that described any changes in GM after consuming KD in neurological patients. Full text of studies such as clinical trials and cohorts were added. The quality assessment of cohort studies was conducted using the Newcastle–Ottawa Quality Assessment Scale and for the clinical trials using the Cochrane Collaboration tool. The search, screening, and data extraction were performed by two researchers independently.

**Results:**

Thirteen studies examining the effects of the KD on the GM in neurological patients were included. Studies have shown that KD improves clinical outcomes by reducing disease severity and recurrence rates. An increase in Proteobacteria phylum, *Escherichia, Bacteroides, Prevotella, Faecalibacterium, Lachnospira, Agaricus,* and *Mrakia* genera and a reduction in Firmicutes, and Actinobacteria phyla, *Eubacterium, Cronobacter, Saccharomyces, Claviceps, Akkermansia* and *Dialister* genera were reported after KD. Studies showed a reduction in concentrations of fecal short-chain fatty acids and branched-chain fatty acids and an increase in beta Hydroxybutyrate, trimethylamine N-oxide, and N-acetylserotonin levels after KD.

**Conclusion:**

The KD prescribed in neurological patients has effectively altered the GM composition and GM-derived metabolites.

**Supplementary Information:**

The online version contains supplementary material available at 10.1186/s12937-023-00893-2.

## Introduction

Various studies have been published on a new therapeutic method for neurological diseases in recent years. According to the articles, neurological diseases such as epilepsy, autism spectrum disorder (ASD), multiple sclerosis (MS), depression, Parkinson's disease (PD), and Alzheimer's disease (AD) are the major cause of disability-adjusted life years (DALYs) and the second leading cause of mortality in the world, as well as one of society's most burdensome diseases. Studies show that neurological disorders have increased in recent years. The total number of deaths (39% increase) and DALYs (27% increase) from all neurological disorders have increased. Although numerous treatments have been suggested for neurological diseases, patients are resistant and do not respond appropriately [1–4].

Several studies have evaluated diets as a treatment/control method for chronic diseases. The consumption of a prudent/Mediterranean-like diet in chronic obstructive pulmonary disease (COPD) patients, Dietary Approaches to Stop Hypertension (DASH) for metabolic and cardiovascular disease, and Ketogenic Diets (KD) in epileptic patients have been evaluated with satisfactory results. KD has been reported to benefit pediatric-resistant epilepsy since the 1920s. This regime usually has a 4:1 ratio of lipid to non-lipid (fat to protein and carbohydrate), consisting of a tightly controlled high-fat, low-protein, and low-carbohydrate diet. According to studies, this effective treatment increases the levels of ketone bodies (KBs), which are anticonvulsants and provide brain energy as alternatives to conventional fuel. Due to KBs' antioxidant and anti-inflammatory properties, they are essential neuroprotective agents. Besides its effects on epilepsy, KD offers many additional benefits, including improved energy, memory, social functioning, quality of life, and reduced negative affect [5–15].

Additionally, studies indicated that the gut microbiota (GM) composition was significantly altered due to KD's low carbohydrate and high-fat content, considerably improving neurological symptoms. After a week of KD treatment in refractory epileptic patients, Xie G.,et al. 2017, observed a reduction in GM abundance, and increased in Bacteroidetes and a reduction in Proteobacteria. They found a reduction in seizure frequency after KD implementation in these patients [5, 7, 8, 10, 11, 16].

Recent studies introduced the term "microbiota-gut-brain axis" as a functional communication between the GM and the nervous system. According to this theory, GM disturbances are thought to be associated with nervous system disorders such as epilepsy, ASD, MS, Alzheimer's disease, and Glucose Transporter 1 Deficiency Syndrome (GLUT1 DS) [1–3, 5, 16, 17].

GM produces chemicals that significantly impact the nervous system, including short-chain fatty acids (SCFAs), nitric oxide, serotonin, and gamma-aminobutyric acid. Human cells can identify these metabolites, which may influence receptors to act or trigger metabolic pathways by resembling host cell products [2–4, 6, 8, 9].

Nowadays, with advances in our knowledge about the significant role of GM in our body and its relation with the nervous system, also the effect of KD on neural function, researchers have started to evaluate their impact on other neurological diseases, especially in those with the most frequencies, burdens, and persistent symptoms and in most cases, there has been a positive change in control of the activity of their diseases. A study by Ferraris C., et al. 2021, reported an improvement in the reduction of seizure attacks and involuntary movements (> 50%) in epileptic patients who underwent KD for about one month [2, 3, 8–10, 12, 15, 17].

We intend to investigate the effect of KD on the composition and functions of GM and its influence on the progression of neurological diseases, given the increasing prevalence and burden of neurological disorders, the variable responses to existing treatment methods, and the growing awareness of how GM plays a critical role in the functioning of the nervous system.

## Materials and methods

All research steps were performed according to the preferred reporting items for systematic review and meta-analysis (PRISMA) guidelines for reporting in this systematic review [18].

### Search strategy

A systematic literature search was conducted on three electronic databases, including PubMed/Medline, Web of Science, and Scopus using standard keywords until December 2022. Also, to complete our search, we used Google Scholar and the reference of articles related to our topic.

The following search terms were used:

"ketogenic diet" OR "low carbohydrate diet "OR "ketosis" OR "exogenous ketones" OR "keto" OR "ketosis diet" OR "ketones") AND ("microbiome" OR "microbiota" OR "intestinal microbiome" OR "intestinal microbiota" OR "intestinal microflora" OR "microflora" OR "intestinal barrier" OR "gut barrier" OR "leaky gut" OR "gut microbiota" OR "gut microbiome" OR "microbiome-derived metabolites" AND" neurology disease" OR" neurological disorder" OR" neurodevelopment disease."

### Study selection

All references were imported to End Note version 9.3.3, and duplicating studies were removed. Two researchers, MM and NL, reviewed and screened titles, abstracts, and full texts according to inclusion and exclusion criteria. After removing duplicated studies and reviewing the literature by two reviewers, any conflicts during screening were discussed and resolved by the senior authors’ opinion. The population of our study is patients with neurological diseases. The ketogenic diet has been given to patients for intervention, and the clinical outcomes and changing GM are evaluated. The primary outcome investigates changes in GM and their metabolites-derived products, and the secondary outcome pursues improvements in neurological symptoms and clinical conditions. Clinical trials and cohort studies are included in this systematic review.

### Inclusion criteria

1. Studies described any changes in GM after consuming KD. 2. The population of interest was patients with neurological diseases who were on KD. 3. Type of study: cross-sectional, case–control, clinical trial, and cohort. 4. Full-text studies were available in English.

### Exclusion criteria

1. Studies that reported GM changes in non-neurological diseases after consuming KD. 2. reviews, commentaries, case studies, animal studies, and letters.

### Data extraction

Data regarding any changes in GM after consuming KD in neurological patients was retrieved.

The following items were extracted:

1. General and methodological characteristics of the cohort studies (first author name, year of publication and country, study population, and study setting, sample size, type of KD, follow-up duration, microbiota analysis method, changes on microbiota, clinical outcomes and quality score).
2. General and methodological characteristics of the clinical trials (first author name, year of publication and country, study population, and study setting, sample size, intervention group, control group, duration, microbiota analysis method, changes on microbiota, clinical outcomes and quality score).

### Quality assessment

All cohort studies were reviewed using the Newcastle–Ottawa Quality Assessment Scale (NOS) for quality assessment [19]. This scale consists of evaluating the methodological quality of the studies in eight items for cohort studies: Selection of participants (maximum four scores), comparability of subjects (maximum two scores), and assessment of outcome (maximum three scores). According to quality assessment scales, after calculating scores for cohort studies, "good quality" studies define as if a study achieves 3 or 4 points in the selection part, AND 1 or 2 points in the comparability part, AND 2 or 3 points in the outcome part. "Fair quality" studies defined as, if a study achieves two scores in the selection part, AND 1 or 2 scores in the comparability part, AND 2 or 3 points in the outcome part. In addition, if a study gets 0 or 1 in the selection part OR 0 score in the comparability part OR 0 or 1 score in the outcome part, it is considered "poor quality."

We assessed the methodological quality of the interventional studies using the Cochrane Collaboration’s tool for assessing the risk of bias for randomized clinical trials (RCTs) and quasi-experimental trials. The Cochrane Collaboration criteria include seven items for selection bias (random sequence generation and allocation concealment), performance bias, detection bias, attrition bias, reporting bias, and other forms of bias. Two authors assessed the quality of included studies. In case of any disagreement, the issue was resolved by the senior authors’ opinion [20, 21].

### Statistical analysis

Due to heterogeneity between studies in outcomes, outcome assessment methods, study design, and setting, the results were synthesized qualitatively, and no meta-analysis has been done.

## Results

### Search results and study selection

**Fig. 1:**
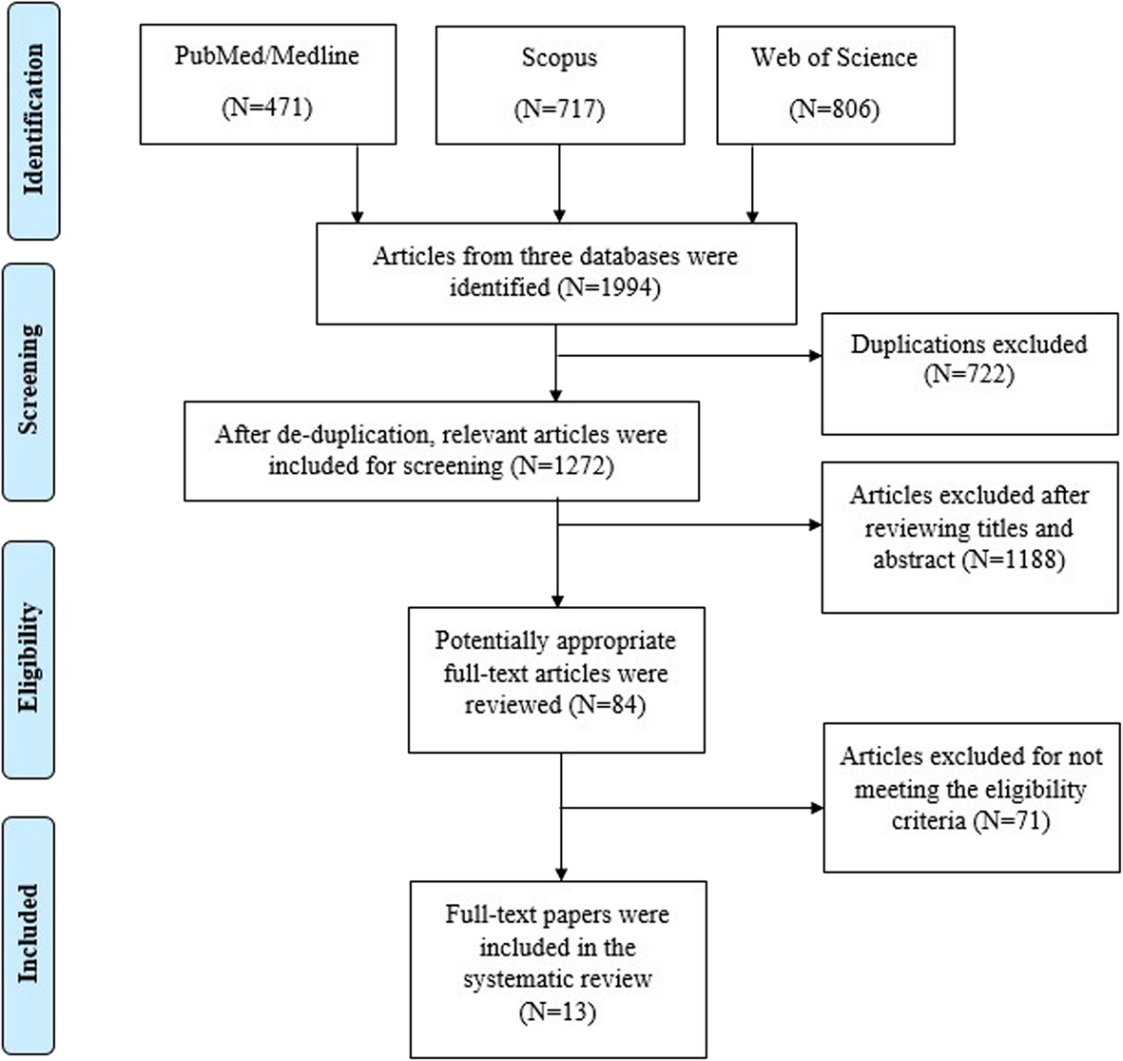
Flow Chart of Study Selection Process

The PRISMA flow diagram for study selection is shown in Fig. 1. In the electronic search of the three databases, 1994 studies were retrieved (PubMed/Medline = 471, Scopus = 717, Web of Science = 806). Seven hundred twenty-two duplicate studies were removed, and 1272 studies remained. After reviewing titles and abstracts, one thousand thirty-eight studies were disqualified. One hundred fifty studies were deprived according to inclusion and exclusion criteria. After that, 84 studies were reviewed for full text. Finally, 13 studies were selected for this systematic review and met the inclusion criteria.

### Study characteristics

Characteristics of the 13 studies eligible for this systematic review are presented in Tables 1 and 2. Of the 13 studies, six (46.2%) were clinical trials [12–17], seven (53.8%) were cohort [5–11], and all studies were published from 2016 until 2022. Follow-up in cohort studies was various, from one month to six months. In clinical trials, studies follow up on KD in patients from one week to eighteen months. Studies were conducted on different continents such as Europe (Italy = 2, Germany = 2, Sweden = 2) [5, 8, 12, 13, 15, 17], America (United States = 4) [6, 9, 11, 14], Asia (Korea = 1, China = 2) [7, 10, 16]. The total number of patients with neurological diseases for this systematic review is 307. In 10 studies, the gender of patients was reported, of which 106 were males, and 63 were females [5, 7–12, 14, 16, 17]. The age of patients varied between studies, and the ages of study participants ranged from 2 to 65 years. Eight studies evaluated KD in children [5, 7–11, 14, 16], and all participants in the three studies were adults [6, 13, 15]. Two studies have been conducted on both children and adults [12, 17].

**Table 1:** General characteristics and significant findings of ketogenic diet in association with gut microbiota and neurological diseases in cohort studies

First author, (Year)	Country	Study population	Sample size	Type of KD	Follow-up duration	Microbiota analysis method	Changes on microbiota	Clinical outcomes	Quality score
Dahlin M., et al. (2022) [5]	Sweden	Children with drug-resistant epilepsy Mean age: 7.7 ± 4.6 yr Male:11 Female:17	*N* = 28	KD	Three months	rDNA sequencing	Increase in *Gordonibacter, Eggerthella lentha, Lactococcus lactis*, *Bifidobacterium longum susp. longum*	Decrease in seizures attacks (> 50%)	Poor
Nagpal R., et al. (2020) [6]	United States	Mild cognitive impairment (MCI) and cognitively normal (CN) Mean age: 64.6 ± 6.4 yr Male: N.A Female: N.A	*N* = 17	MMKD	Six weeks	Sequencing of the fungal rRNA ITS1 gene	Increase in *Agaricus*, *Mrakia*, decrease in *Saccharomyces*, *Claviceps*	Positive affect on cognitive health	Good
Lee K., et al. (2020) [7]	South Korea	Children with epilepsy Mean age: 3.17 yr Male: 3 Female: 5	*N* = 8	KD	One month	16S rDNA sequencing	Decrease in *Enterococcis faecium, Bifidobacterium longum, Eggerthella lenta*	Decrease in seizures attacks (> 50%)	Good
Lindefeldt M., et al. (2019) [8]	Sweden	Children with drug-resistant epilepsy Mean age: 7.6 ± 4.5 yr Male:10 Female:14	*N* = 24	KD	Three months	rDNA sequencing	Decrease in *Bifidobacteria E. rectale*, *Dialister*, increased in *E. coli*	Decrease in seizures attacks (> 50%), improved cognition and motor functions	Good
Mu C.,et al. (2019) [9]	United States	Children with ASD and TC Mean age in ASD: 9 ± 2.17 yr Mean age in TC: 11 ± 4.13 yr Male:24 Female:3	*N* = 27	Modified KD ( a gluten free diet incorporating MCT oil)	Threemonths	Gas Chromatography-Mass Spectrometry, H Nuclear Magnetic Resonance Spectroscopy, Inductively Coupled Plasma-Mass Spectrometry	Increase in gut microbe-derived trimethylamine N-oxide	Negative correlation between selenium and behaviour scores, negative correlation between ornithine and ADOS-2 Overall Score and Social Affect Score	Good
Zhang Y., et al. (2018) [10]	China	Children with refractory epilepsy Mean age: 4.2 yr Male:14 Female:6	*N* = 20	KD	Six months	16S rDNA sequencing	Decrease in *Firmicutes*, increase in *Bacteroidetes*	Decrease in seizures attacks (≥ 90%) in 3 patients, decrease in seizures attacks (50 to 89%) in 5 patients, decrease in seizures attacks (< 50%) in 10 patients, seizure free in 2 patients	Poor
Spinelli E., et al. (2018) [11]	United States	Children with epilepsy Mean age: 4.2 yr Male:14 Female:6	*N* = 20	KD	Six months	16S rDNA sequencing	Decrease in *Firmicutes*, *Actinobacteria,* increase in *Bacteroides,* increased in *Clostridiales, Clostridia, Ruminococcaeceae, Lahnospiraceaea, Alistipes*, *Tikenellacase*	Decrease in seizures attacks (≥ 50%) in responder patients	Poor

*KD* Ketogenic diet, *ASD* Autism spectrum disorders, *TC* Typically development control, *MCT* Medium-chain triglyceride, *MCI* Mild cognitive impairment, *CN* Cognitively normal, *DNA* Deoxyribonucleic acid, *MMKD* Modified Mediterranean ketogenic diet

**Table 2:** General characteristics and significant findings of ketogenic diet in association with gut microbiota and neurological diseases in clinical-trial studies

First author, (Year)	Country	Study design	Study population	Sample size	Intervention group	Control group	Duration	Microbiota analysis method	Changes on microbiota or metabolites derive	Clinical outcomes
Ferraris C., et al. (2021) [12]	Italy	Single-arm, uncontrolled, quasi experimental	Patients with epilepsy Age: 2-46 yr Male:3 Female:4	*N* = 7	*N* = 7	N.A	One month	Cytotoxicity, genotoxicity, measuring SCFA	Decrease in fecal SCFA, (acetate, propionate, butyrate), isobutyrate	Decrease in seizures attacks (> 50%) and involuntary movements
Bahr L., et al. (2018) [22]	Germany	Single-center, randomized, controlled, parallel-group study	Patients with relapsing–remitting MS Age: 18-65 yr Male: N.A Female: N.A	*N* = 111 (37 patients in each KD, FD and SD groups)	*N* = 74 (37 patients in KD group and 37 patients in FD group)	*N* = 37	Nine months	Biochemical measures	Increase in serum beta hydroxybutyrate level	Improve cognition in KD group
Lee R., et al. (2018) [14]	United States	Open-label, observer-blinded, quasi experimental	children with ASD Mean age: 7.9 ± 3.3 yr Male:13 Female:2	*N* = 15	*N* = 15	N.A	Three months	Biochemical measures	Increase in serum beta hydroxybutyrate level	Improve behaviors
Swidsinski A., et al. (2017) [15]	Germany	Controlled, randomized clinical trials	Patients with MS Mean age: N.A Male: N.A Female: N.A	*N* = 24 (10 patients in KD and 14 patients in control group)	*N* = 10	*N* = 14	Six months	FISH with ribosomal RNA derived probs	Decrease in pioneer bacterial groups, increase in *Akkermansia*	Positive effects on MS patients
Xie G., et al. (2017) [16]	China	Controlled, randomized clinical trials	Children with epilepsy Mean age: 1.95 ± 3.1 yr Male: 11 Female: 3	*N* = 44 (14 patients in KD group and 30 healthy infants in control group)	*N* = 14	*N* = 30	One week	16 s rRNA sequencing	Decrease in *Proteobacteria Cronobacter,* no change in *Bacteroides,* increase in *Prevotella*, *Bifidobacterium*	Clinical improvement (64%), decrease in seizure frequency (50%)
Tagliabue A., et al. (2016) [17]	Italy	Prospective, single-center, single-arm quasi experimental	Epileptic patients affected by GLUT1 DS Age: 8-34 yr Male:3 Female:3	*N* = 6	*N* = 6	N.A	Three months	RT-PCR and bacterial DNA extraction	Increase in *Desulfovibrio spp*.	No change

*KD* Ketogenic diet, *FD* Fasting diet, *SD* Standard diet, *MS* Multiple Sclerosis, *SCFA* Short chain fatty acid, *FISH* Fluorescence in situ hybridization, *RT-PCR *Real time polymerase chain reaction, *GLUT1DS* Glucose transporter type 1 deficiency syndrome, *RNA* Ribonucleic acid, *DNA* Deoxyribonucleic acid, *ASD *Autism Spectrum disorder

### Quality of studies

The quality assessment of cohort studies was conducted according to the Newcastle–Ottawa Quality Assessment Scale. In this systematic review, four cohort studies were assessed as good qualities [6–9] and three as poor qualities [5, 10, 11]. Because the non-exposure group was not considered in these three studies and the patients were evaluated before and after the KD treatment period, therefore they obtained poor quality in design. All six clinical trials were described as low-risk bias studies in performance, detection, and reporting biases. Four studies (66.6%) were determined as high risk for attrition bias; two expressed low risk in this category. Regarding the selection bias, three studies (50%) were considered as high-risk bias studies due to being quasi-experimental. All studies had an unclear risk for other biases (Supplementary Tables 1 and 2).

### Clinical outcomes

Most studies show that KD improved clinical outcomes by reducing disease severity, attack frequency, and relapse rate.

KD's effect on children with refractory epilepsy has been evaluated in seven studies. In these studies, seizure frequencies decreased by more than 50% with concomitant electroencephalogram (EEG) improvements. One study examined the effect of KD on refractory epilepsy at all ages. Moreover, these studies indicate that KD reduced seizure frequency and improved treatment response in most patients. They also found that KD significantly shortened the attack duration and post-ictal phase. In some cases, a complete response was observed, and patients were free of seizures. They also observed decreased post-ictal fatigue, improved cognition, and improved motor function in some patients [5, 7, 8, 10–12, 16].

GLUT-1 DS patients' paroxysmal dyskinesia and progressive global resistance to physical exertion were eliminated following KD treatment in one study [17]. Two studies show that KD and MS treatment responses correlate positively. The patient's quality of life improved during KD treatment, and the relapse rate, disability, fatigue, and depression were reduced [13, 15].

KD has been evaluated in two studies on children with ASD. To assess KD intervention clinical outcomes, the autism diagnostic observation schedule 2nd Edition (ADOS-2) and childhood autism rating scale 2nd Edition (CARS-2) were used. These studies indicate that most KD patients have higher ADOS-2 and CARS-2 scores than their peers. Social, behavioral, and comparison scores developed during treatment, and one study found a significant relation between KD and CARS-2 scores [9, 14]. Moreover, in one study on patients with mild cognitive impairment (MCI), KD positively affects cognitive impairment by normalizing and balancing the GM [6].

### Taxonomic changes in the GM

Six studies examined the changes in GM after KD in patients with refractory seizures [5, 7, 8, 10, 11, 16]. Dahlin M. et al. 2022 evaluated KD efficiency in drug-resistant epileptic patients and its effect on GM. The study found that the bacteria *Gordonibacter pamelaeae, Eggerthella lentha, Lactococcus lactis,* and *Bifidobacterium longum susp. Longum* were associated with anti-seizure responses, while others, *Alistipes shahii* and *Eubacterium rectale*, related with no reaction to KD. The authors suggest that specific *Bifidobacteria* may reduce seizures in individuals with refractory epilepsy [5]. KD treatment reduced species diversity, increased *Bacteroides*, and decreased *Firmicutes* and *Actinobacteria* after a study conducted by Spinelli E. et al. 2018. The prevalence of *Clostridiales, Clostridia, Ruminococcaeceae, Lahnospiraceaea, Alistipes,* and *Tikenellacases* was significantly higher in non-responders to KD treatment. After implementing KD, some changes in GM composition and a 50% reduction in seizure frequency in patients were observed. The clinical improvement could be related to modulation of GM or not and further investigations are needed [11].

A significant increase in Proteobacteria and the genus Escherichia and a reduction in *Actinobacteria* (primarily due to a reduction in the genus *Bifidobacterium*) after three months of KD treatment in severe epileptic patients were observed by Lindefeldt M. et al.2018. In addition to the decrease in *Eubacterium rectale* and *Dialister*, 41.7% of patients experienced a 50% reduction in seizures. 83.3% of the patients reported improvement in their cognition and motor functions [8].

Based on Zhang Y. et al. 2018, there was a significant reduction in Alpha diversity after KD treatment. *Bacteriodetes* levels were significantly increased, and *Firmicutes* levels decreased. Non-responsive patients had increased levels of *Clostridiales, Ruminococcaceae, Rikenellaceae, Lachnospiraceae,* and *Alistipes*. A total of 10% of the patients became seizure-free, 15% experienced a reduction of seizures exceeding 90%, 25% experienced a reduction of 50 to 89%, and 50% experienced a drop of less than 50%. Electroencephalograms (EEGs) were improved in all patients, with a more than 50% reduction in seizures [10].

According to Xie G. et al. 2017, *Bacteroides* and *Prevotella* levels increased significantly, whereas *Cronobacter* levels decreased. Approximately 21% of participants became seizure-free, and 43% experienced a 50% to 90% reduction in seizure frequency. Other pathogens mentioned include *Streptococcus, Alistipse, Ruminiclostridium, Barnesiella, Enterococcus,* and *Erysipelatoclostridium* also decreased after KD [16].

In a study by Lee R. et al.2018, they found decreased levels of *Bifidobacterium, Eggerthella*, and *Enterococcus* while increasing levels of *Bacteroides, Faecalibacterium, Lachnospira, Roseburia,* and *Veillonella* [14].

According to one study, there was an increase in *Agaricus* and *Mrakia* genera after KD treatment in MCI patients, while *Saccharomyces* and *Claviceps* (higher classification) decreased [6].

A study compared GM changes in MS patients after KD. The authors noted a reduction in pioneer bacterial groups and an increase in *Akkermansia* genus concentrations [15].

KD has been shown to affect GM in GLUT-1 patients in one clinical trial. This study reported an increase in *Desulfovibrio spp.* [17].

### Changes in GM-derived metabolites

The effects of KD on GM-derived metabolites in patients with refractory seizures were examined in one study; this study demonstrated a decrease in fecal SCFA concentrations, including acetate, propionate, butyrate, and branched-chain fatty acids, with a positive relation between isobutyrate and changes in GM composition [12].

KD decreased beta Hydroxybutyrate (BHB) serum level as a gut-derived metabolite in patients with ASD [14]. A cohort study reported increased GM-derived trimethylamine N-oxide and N-acetylserotonin levels [9]. A clinical trial study evaluated the KD treatment on MS patients and observed a high serum level of BHB after nine months of treatment [13].

## Discussion

Several studies have demonstrated that the KD confers neuroprotection by restoring/promoting beneficial microbes in the GM of patients with neurodegenerative diseases. In addition, this regime can potentially improve and regulate memory, learning, and disease progression, reducing the frequency of relapses and attacks. During the KD regime, GM compositions and GM-derived metabolites were replaced, these changes possibly result in clinical improvements [1–4, 23–27].

### Effect of the ketogenic diet on neurological diseases through ketone bodies

Although the underlying pathology of numerous neurological diseases has not been entirely determined, the role of inflammation, oxidative stress, and mitochondrial dysfunction in some neurological diseases, including Seizure, MS, ASD, PD, and AD has been identified. KBs produced from KD implementation can provide neuroprotective effects, including reducing oxidative stress, sustaining energy levels for CNS cells, adjusting deacetylation activity, and modulating inflammatory responses [28].

Several neuronal injuries result from glutamate excitotoxicity, calcium overload, mitochondrial dysfunction, and oxidative stress. The ability of KBs to counteract oxidative stress has been observed in studies, particularly in protecting the nervous system. Mitochondria is known to be the main source of reactive oxygen species (ROS) production, and glutathione peroxidase (GSH-Px) is an important enzyme involved in ROS formation process. In normal condition, superoxide anion production during oxidative phosphorylation is relatively low. However, when mitochondria are damaged, calcium ions become overloaded, and ROS level increases, leading to excitotoxic damage [29, 30]. KD helps lower blood glucose levels and promotes ketone production in the liver. The increase in KBs primarily occurs through the oxidation of fatty acids, particularly polyunsaturated fatty acids (PUFAs). PUFAs activate peroxidase by blocking voltage-gated sodium and calcium channels and regulating membrane receptors in neurons or inducing the expression of mitochondrial uncoupling protein (UCP). This uncoupling process reduces mitochondrial membrane potential, ultimately decreasing ROS production [29, 31]. The oxidative regulation has an impact on Complex I/III in the ROS/RNS respiratory chain. Research has shown that mitochondrial dysfunction and the inhibition of complexes I, II, and III can occur due to epileptic seizures. However, using KD can enhance the inhibition of complex II/III and significantly improve mitochondria function during oxidative stress [28, 32, 33]. Although the exact mechanisms by which KD reduces seizures are not fully understood, KBs and PUFAs, which can be increased through KD implementation and GM alterations, may play critical roles in its anti-seizure effects. As stated in the studies, KBs increase inhibitory neurotransmitters, activate potassium channels, and enhance energy production in the nervous system, thereby raising the seizure threshold in the brain, and PUFAs lead to increased energy transcripts, enhanced energy reserves, and stabilized synaptic function. This ultimately prevents neuronal hyperexcitability, which leads to anti-seizure function [34].

As mentioned above, KD has the potential to improve the functioning of mitochondria and alter glucose metabolism, leading to a decrease in the production of advanced glycation end products (AGE). The accumulation of AGE during the aging process can speed up the progression of AD. KBs, particularly β-Hydroxybutyric acid (βHB), have been found to mitigate the toxicity of 1-methyl-4-phenylpyridine (MPP +) on neurons cultured in vitro and reduce the toxicity of amyloid protein fragment (Aβ) on hippocampal neurons [35]. Additionally, according to the animal study of Beckett T, et al. 2013, KD can enhance the electrophysiological function of the brain in AD mice [36]. While animal studies have shown promising results, clinical research has not yet provided definitive conclusions.

### Effect of the ketogenic diet on neurological diseases through gut microbiota

Recent studies suggest complex interactions between the GM and the central nervous system (CNS). The GM influences the development and balance of the CNS through immune, circulatory, and neural pathways, while the CNS affects the GM through stress and endocrine responses, called the "gut microbiota-brain axis." The vagus nerve is mainly responsible for the direct communication between GM and CNS. As stated in the studies, cutting this nerve reduced neurogenesis regulated by the GM and expression of brain-derived neurotrophic factor in the hippocampus [37]. Moreover, the GM also produces neurotransmitters and neuropeptides. *Enterococcus spp., Streptococcus spp*., and *Escherichia spp.* generate serotonin; *Lactobacillus spp. and Bifidobacterium spp.* produce gamma-aminobutyric acid (GABA); *Escherichia spp.,* and *Bacillus spp.* produce noradrenaline and dopamine. Some species in Bacteroidetes and Firmicutes phylum produce short-chain fatty acids (SCFAs) like acetate, propionate, and butyrate through the fermentation of insoluble dietary fibers. GM produces an enzyme called glutamate decarboxylase, which converts glutamate to GABA. These bacteria have also been shown to affect the expression of GABA and the N-methyl-D-aspartate (NMDA) receptors in the brain in animal models [37]. In animal studies, it has been shown that modulating gut microbiota composition could be effective on neurotransmitters’ concentration. For example, when mice were given *Lactobacillus rhamnosus* orally over a long period, it led to increased expression of GABAB1b mRNA in the cingulate and prelimbic region and accompanying reduced expression in the hippocampus, amygdala, and locus coeruleus. These neurotransmitters cannot cross the blood–brain barrier (BBB) and have limited direct effect on CNS function; however, they may indirectly influence the CNS system through the enteric nervous system, vagus nerve, and modulation of peripheral receptor expression. Moreover, imbalances in the GM can lead to increased intestinal barrier permeability and activation of an immune response in peripheral tissues. This can result in heightened signalling of cytokines/chemokines through neuronal or humoral pathways, potentially triggering an inflammatory response in the CNS where disruption of the BBB is considered an essential step [37, 38]. The study by Olson S., et al.2018, declared that KD alters GM and can protect against acute seizures in a mouse model. Mice treated with *Akkermansia* and *Parabacteroides* were protected against seizures compared to those in the control diet group [39].

### Taxonomic changes in the GM and clinical outcome

It has been extensively studied that KD is effective in treating patients with severe and refractory seizures, and fortunately, the results of various studies have been admissible. The effects of GM changes on clinical improvement in epileptic patients treated with KD were examined in six studies. Since the KBs produced in the KD, as a source of energy for the brain, can pass through the blood–brain barrier by a special blood transporter, the issue of improvement of patients' symptoms using this diet was investigated. Various studies have explored its effects on the amelioration of patients' treatment [25–27, 40–47].

Although the difference in the amount of intervention period and follow-up time was different in several studies, overall, the changes in the composition of GM were in favor of an increase in the relative abundance of bacterial genera Escherichia (*E. coli*), Clostridia (*Clostridiales, Clostridium, Lachnospiraceae,* and *Ruminococcaceae*), *Alistipes*, *Bacteroides*, *Desulfovibrio*, Actinomycetaceae family and Bacteroidetes phylum and a decrease in the relative abundance of bacterial genera, *Bifidobacteria* (*B. longum*), Eubacterium (*E. rectale*), *Dialister*, Enterococci (*E. faecium*), Eggerthella *(E. lenta*), *Cronobacter*, and some genera of *Firmicutes* and Proteobacteria phylum were observed. As stated in these studies, more than a 50% reduction in patient seizure attacks was reported. This clinical improvement could be a result of these new GM combinations after KD treatment.

Unfortunately, most studies have had small sample sizes because KD is an expanding but uncommon treatment option in neurodegenerative diseases. The studies had varied designs, sample processing, analysis methods, and demographic characteristics. Therefore, a lack of consistent outcomes is expected. For example, two cohort studies and one clinical trial confirmed that *Bifidobacteria* abundance was reduced in drug-resistant epilepsy patients after ingestion of KD. This finding is not surprising since KD is usually fiber-free, and *Bifidobacteria* needs fiber to survive [5, 8, 16]. However, in a cohort study, the genus *Bifidobacteria* was increased in epileptic children compared with healthy age-matched controls after KD treatment [7]. Therefore, the results of studies regarding changes in *bifidobacteria* abundance were conflicting.

Tumor Necrosis Factor Alpha (TNF-α) is an inflammatory cytokine associated with epilepsy. In addition, *Bifidobacteria* species (*B. longum and B. breve*) were associated with TNF-α levels, and they were higher in patients who started KD and, they had experienced a reduction in seizure frequency. *Bifidobacteria* interact with the immune system through TNF-α, affecting the seizure threshold. *Bacteroides* has a role to digest and metabolize high-fat food and to regulate the secretion of IL-6 and IL-17 in dendritic cells (DCs), a process strongly associated with seizure severity of epileptic patients. Reduction in the Firmicutes, along with an increased level of the genus *Bacteroides*, is also related to the high production of SCFAs, which have antiepileptic effects [5, 8, 16].

Concerns about the increasing incidence of degenerative diseases such as AD and MCI, their irreversible complications, and the disproportionate response to standard treatments have led to examining another treatment method, including the KD, in these patients. One study evaluated this diet for GM changes and clinical improvement in patients. In the study by Nagpal R. et al. 2020, MCI patients had a higher percentage of fungal families such as *Sclerotiniaceae*, *Phaffomyceteceae*, *Trichocomaceae*, *Cystofilobasidiaceae*, *Togniniaceae*, and genera such as *Botrytis*, *Kazachstania*, *Phaeoacremonium*, and *Cladosporium*. In contrast, the control group had fewer *Cladosporiaceae* and *Meyerozyma*. After KD treatment, an increase in *Agaricus*, *Mrakia*, and a reduction in *Saccharomyces* and *Claviceps* were reported [6].

Fungi and bacteria coexist symbiotically in the human gut, and their interactions can be impaired in the disease states. According to the studies, a complex ecological co-regulatory network between them exists in a healthy person, which is disturbed in MCI. Different fungi play an essential role in the GM community stability and function, as seen in patients with MCI. Fungi like *Meyerozyma*, *Wallemia*, and *Aspergillus* correlate with several bacterial species in Firmicutes phylum and *Bacteroides*, *Roseburia*, and *Eubacterium* genera [3, 4, 6, 41–43, 48–57]. These data suggest that the KD modulates the fungal composition of the gut, which can influence the GM and the GM-derived metabolites.

In addition to the many treatment challenges associated with MS, immunomodulatory medications are the only treatments available to slow the disease's progress. Many studies have reported that MS patients have an underlying dysbiosis caused by reduced biodiversity and concentrations of essential bacterial groups, such as *Faecalibacterirum prausnitzii*. According to the study by Swidsinski et al. 2017, KD's effect on GM was biphasic. As mentioned in the study, first, bacterial diversity and concentration were reduced. After that, gut bacteria were restored at the end of the 12-week treatment period, and over time, they overpassed the baseline values [15, 27–39, 58–63].

An inherited disease known as GLUT1 DS disrupts glucose transport as a fuel supply for the brain, leading to seizures, impaired neurological development, and movement disorders. According to Tagliabue A. et al. [17], a survey was conducted on these patients evaluating KD's effects on GM. After three months of KD, *Desulfovibrio spp.* increased significantly. All patients experienced relief from paroxysmal dyskinesia and progressive global resistance to physical effort [17]. The results are preliminary in GLUT1 DS and MCI studies. Therefore, further studies must be conducted to prove and corroborate the results [6, 17].

### Changes in GM-derived metabolites and clinical outcomes

Following KD, we observed reduced fecal SCFA concentrations, including acetate, propionate, butyrate, and branched-chain fatty acids, and increased BHB, trimethylamine N-oxide, and N-acetylserotonin [9, 12–14].

The KD's impact on the GM community alters the GM composition and metabolites. In several studies, metabolite changes have been examined, and their impact on neurological disease progression has been reviewed. Ferraris C. et al. 2021 read the effect of KD on GM-derived metabolites after a one-month KD diet treatment. Similar to this study, studies regarding GM in neurological disorders have also reported significant decreases in fecal SCFA concentrations, such as acetate, butyrate, propionate, and iso-butyrate [12, 39–51, 63–67].

ASD is a neurodevelopmental disorder characterized by multiple impairments in social interaction, repetitive behaviors, and interpersonal communication. ASD is associated with metabolism dysregulation and disruption of immune function, according to studies. Researchers have also investigated the effects of KD on ASD patients. In a three-month pilot study conducted by Mu C. et al. 2019, KD was examined for its impact on these patients. In their research, ketones and other metabolites, including 3-hydroxybutyrate, acetoacetate, acetone, and acetylcarnitine, increased their relative concentrations. Amino acid concentrations decreased, including glutamine, tyrosine, phenylalanine, histidine, and alanine. KD treatment resulted in a significant reduction in chromium levels and an increase in nickel and selenium levels. There was a significant negative correlation between acetoacetate and the comparison score. The ADOS-2 overall score was negatively correlated with the social effect score, whereas chromium and creatine were positively correlated with the comparison score. N-acetyl serotonin negatively correlates with behavioral index, and acetone negatively correlates with social affect scores. Lee R. et al. 2018, also assessed KD in ASD patients. Their findings showed that BHB serum levels significantly increased after three months of KD treatment. Based on the ADOS-2 score, they observed significant improvement in core autism characteristics. There were also substantial advancements in CARS-2 items related to imitation, body use, and fear or nervousness. There was no significant difference in restricted and repetitive behavior scores between patients [9, 14, 45, 47, 52–59, 68–76]. These two studies (one clinical trial and one cohort study) reported an increase in serum levels of BHB in ASD patients. Since both of these studies were conducted on children with ASD, the consistency of the results, despite the difference in the study design, is considerable [9, 14].

It is believed that the ketogenic state exerted by the KD in the presence of low carbohydrates promotes positive modulation, which increases and preserves brain function. Furthermore, a positive change has occurred due to the KD, replacing bacteria with anti-inflammatory and supportive effects. For instance, in the study conducted by Swidsinski et al., 2017, a decrease in the concentration of *Faecalibacterirum prausnitzii* as one of the reasons for dysbiosis was observed in MS patients. By secreting butyrate, *F. prausnitzii* promotes the preservation and maintenance of regulatory T cells and T helper 17 cells, preventing inflammation. Studies conducted on neurological disorders demonstrated an increase in *F. prausnitzii* concentration and clinical improvements after KD consumption [3, 4, 13, 15, 22, 71–76].

A strength of this study is that we included both cohort and clinical trial studies, which were conducted on all age groups, making it comprehensive. This study has some limitations too. Since KD application to neurological disorders is a relatively new topic, few studies have been conducted in this field. Due to the small number of studies and heterogeneity in study designs and results, conducting a meta-analysis is impossible. In addition, most studies have been conducted on a limited number of patients for a short period. There have been studies in which patients started the KD regime along with their medications; as a result, it has been challenging to compare the effects of this regimen alone. Further studies with more patients and longer treatment and follow-up periods are warranted. Age-matched control groups are also recommended in these studies.

## Conclusion

Neurological diseases can ultimately affect human health through multiple mechanisms, including oxidative damage, energy metabolism disorders, or inflammatory reactions. The use of the ketogenic diet in treating neurological diseases has been noticed recently. Its effects have been proven, especially in the treatment of drug-resistant seizures. Based on studies, these positive effects are due to the role of this regimen in the alteration of GM composition and their metabolites. In these studies, we found an increased in Proteobacteria*, Bacteroides, Escherichia, Prevotella, Faecalibacterium, Lachnospira, Agaricus,* and *Mrakia* genus and a reduction in Firmicutes, Actinobacteria, *Eubacterium rectale, Cronobacter, Saccharomyces, Claviceps, Akkermansia* and *Dialister* were reported. In addition, we noticed the reduction of fecal SCFA concentrations, including acetate, propionate, butyrate, and branched-chain fatty acids, and increased serum levels of BHB, trimethylamine N-oxide, and N-acetylserotonin after KD. The efficacy of KD in reducing relapse and developing diseases has been reported, and all studies stated improvement in clinical outcomes after the diet and modulation of composition and function of GM is considered as one of the underlying mechanisms of KD. The detailed mechanisms of KD for treating neurological diseases remain unclear; in some neurological diseases, such as epilepsy, AD, and PD, it can have a therapeutic effect. Conversely, it has a supporting role in other diseases by helping to treat the disease and improve the patients' symptoms and quality of life. KD demonstrates excellent potential in clinical application, but further exploration is needed. Future studies must elucidate the role of components in KBs and their therapeutic targets and related pathways to optimize the strategy and efficacy of KD treatment in neurological diseases.

### Supplementary Information

**Additional file 1: Supplementary Table 1. **Newcastle-Ottawa Scale Adapted for Cohort Studies.

**Additional file 2: Supplementary Table 2. **The Cochrane Collaboration's tool for assessing risk of bias in clinical trial study.

## Data Availability

Not applicable.
