# Supplementary material for: Does the ketogenic diet improve neurological disorders by influencing gut microbiota? A systematic review
Source: Nutr J. 2023 Nov 20;22:61. doi: 10.1186/s12937-023-00893-2 (PMC10658738; doi:10.1186/s12937-023-00893-2)
Supplement: Supplementary file 1 — Additional file 1: Supplementary Table 1. Newcastle-Ottawa Scale Adapted for Cohort Studies. [file 12937_2023_893_MOESM1_ESM.docx]

**Supplementary Table 1. Newcastle-Ottawa Scale Adapted for Cohort Studies**

| First author (Year) | Study design | Selection | | | | Comparability | Outcomes | | | Quality score |
| --- | --- | --- | --- | --- | --- | --- | --- | --- | --- | --- |
|  |  | Representativeness of the exposed | Selection of the non-exposed | Ascertainment of exposurents | Demonstration that outcome |  | Assessment | Follow-up | Median duration of follow-up |  |
| Dahlin M., et al.  (2022) | Cohort study | 1 | 0 | 1 | 1 | 0 | 1 | 1 | 1 | Poor |
| Nagpal R., et al.  (2020) | Cohort study | 1 | 0 | 1 | 1 | 1 | 1 | 0 | 1 | Good |
| Lee K., et al (2020) | Cohort study | 1 | 1 | 1 | 1 | 1 | 1 | 0 | 1 | Good |
| Lindefeldt M., et al. (2019) | Cohort study | 1 | 0 | 1 | 1 | 1 | 1 | 1 | 1 | Good |
| Mu C.,et al. (2019) | Cohort study | 1 | 1 | 1 | 1 | 1 | 1 | 1 | 1 | Good |
| Zhang Y., et al. (2018) | Cohort study | 1 | 0 | 1 | 1 | 0 | 1 | 1 | 1 | Poor |
| Spinelli E., et al. (2018) | Cohort study | 1 | 0 | 1 | 1 | 0 | 1 | 1 | 1 | Poor |
