# Supplementary material for: Does the ketogenic diet improve neurological disorders by influencing gut microbiota? A systematic review
Source: Nutr J. 2023 Nov 20;22:61. doi: 10.1186/s12937-023-00893-2 (PMC10658738; doi:10.1186/s12937-023-00893-2)
Supplement: Supplementary file 2 — Additional file 2: Supplementary Table 2. The Cochrane Collaboration's tool for assessing risk of bias in clinical trial study. [file 12937_2023_893_MOESM2_ESM.docx]

**Supplementary Table 2. The Cochrane Collaboration's tool for assessing risk of bias in clinical trial study.**

| Ferraris C., et al. 2021 |  |  |  |  |  |  |
| --- | --- | --- | --- | --- | --- | --- |
| Bahr L., et al. 2018 |  |  |  |  |  |  |
| Lee R., et al. 2018 |  |  |  |  |  |  |
| Swidsinski A., et al. 2017 |  |  |  |  |  |  |
| Xie G., et al. 2017 |  |  |  |  |  |  |
| Tagliabue A., et al. 2016 |  |  |  |  |  |  |

Attrition bias

Reporting bias

Other biases

Detection bias

Performance bias

Selection bias

**Key**

High risk of bias

Unclear risk of bias

Low risk of bias
